# Parents’ Attitudes toward Childhood Vaccines and COVID-19 Vaccines in a Turkish Pediatric Outpatient Population

**DOI:** 10.3390/vaccines10111958

**Published:** 2022-11-18

**Authors:** Nihal Durmaz, Murat Suman, Murat Ersoy, Emel Örün

**Affiliations:** 1 Department of Pediatrics, Gulhane Training and Research Hospital, Ankara 06010, Turkey; 2 Department of Pediatrics, Afyon Çay State Hospital, Afyon 03700, Turkey; 3 Department of Pediatrics, Mersin City Hospital, Mersin 33330, Turkey; 4 Department of Pediatrics, Ankara Liv Hospital, Ankara 06680, Turkey

**Keywords:** vaccine hesitancy, childhood vaccinations, COVID-19, Turkey

## Abstract

Vaccination hesitancy (VH) is an important public health issue. The determinants of parental decisions on whether to vaccinate their children are multidimensional and need to be carefully considered in the COVID-19 era. Our study aims to investigate the prevalence of VH among parents, parents’ use of social media, and their attitudes toward the COVID-19 vaccine upon vaccine refusal. Materials and methods: Our participants were the parents of children admitted to hospitals in three different cities in Turkey between September 2021 and December 2021. The parents were asked to complete sociodemographic data and their attitudes toward COVID-19 diseases, the Parental Attitudes Toward Childhood Vaccines (PACV) scale, and the Attitudes Toward COVID-19 Vaccine (ATV-COVID-19) scale. Participants were categorized as “non-hesitant”, with a score of <50, and “hesitant”, with a score of ≥50. Results: A total of 1087 parents with a mean age of 33.66 (SD 9.1) years old participated in the study. VH was noted in 102 (9.38%) parents. Age, gender, education, and income levels did not significantly differ from one another, according to the PACV; however, parents who delayed vaccinating their children and indicated that social media had an impact on vaccination decisions were more hesitant. Parents who were male and had a family member diagnosed with COVID-19 showed more positive attitudes in the ATV-COVID-19. Parents who were hesitant about childhood vaccinations had lower positive attitudes toward the COVID-19 vaccine (2.84 ± 0.97) than parents who were not hesitant (3.77 ± 0.9). A total of 761 (70.14%) parents need more information about childhood immunizations. Conclusion: Parents who are hesitant about childhood immunization programs in Turkey have a less positive attitude toward COVID-19 vaccines and are affected by social media. Parents need information about vaccines, and because the controversy surrounding COVID-19 vaccines can diminish parents’ confidence in routine childhood immunizations, understanding the complex causes behind vaccination hesitancy can help public health policy break through barriers and increase immunization rates.

## 1. Introduction

Vaccine hesitancy (VH) refers to a delay in accepting or refusing vaccination despite the availability of immunization services, as defined by the Strategic Advisory Group of Experts that work for the WHO [1]. Although VH has been known since the era of widespread vaccine use, it has gained increased acceptance in the last two decades [2]. The need for vaccines and the safety of vaccines are now being questioned as mortality and morbidity rates from vaccinations have declined. For this reason, some people are reluctant to be vaccinated and occasionally choose not to be vaccinated [3]. Vaccination decisions are influenced by a complex interplay of social, psychological, spiritual, political, and personal factors. Furthermore, questioning vaccines and reluctance to vaccinate are exacerbated by social media platforms [4].

Social media and the Internet have become an indispensable part of health-related information behavior today, and the rapid dissemination of health information via social media is a public health opportunity [5]. The Internet and social media are very active in spreading information that questions the safety and efficacy of vaccines, and it is difficult to change the negative attitudes of people exposed to false information about vaccines [4,5]. Some platforms, such as Facebook, Instagram, YouTube, and Twitter, have taken various enforcement actions by removing posts or suspending accounts to prevent the spread of vaccine misinformation [6,7].

In Turkey, vaccine hesitancy–refusal has increased over the past decade. Previously, cases of vaccination refusal were very rare but have increased rapidly since 2015 after the victory of a lawsuit for “obtaining parental consent for vaccination”, as well as frequent arguments against vaccination in the media. The number of families who refused to vaccinate their children was 183 in 2011, which increased to 980 in 2013, 5400 in 2015, 12,000 in 2016, and 23,000 in 2018 [8].

The aim of our study is to investigate the prevalence of VH and the attitudes of parents toward the COVID-19 vaccine and related barriers and facilitators. In addition, our study aims to evaluate the association between parents’ use of social media and their attitudes towards the COVID-19 vaccine and vaccine hesitancy. 

## 2. Materials and Methods

### 2.1. Study Design, Setting, and Participants

This cross-sectional study was prepared according to the Strengthening the Reporting of Observational Studies in Epidemiology (STROBE) guidelines [9]. The study was conducted with parents of children aged 0–60 months. The study was created with convenience samples obtained from three different hospitals: Gulhane Training and Research Hospital; Mersin City Training and Research Hospital, Department of Pediatrics; and Afyon Çay State Hospital. The research was completed between September 2021 and December 2021. The first two hospitals were second- and third-level care hospitals, while the other was a primary care hospital. General pediatric outpatient clinics in hospitals in Turkey provide primary care services to pediatric patients who are between 0–18 years old. These hospitals are by direct referral and do not require a referral chain; a general pediatrician may refer the patient to a pediatric subspecialist when deemed necessary. Patients who were being treated for chronic diseases and presented to outpatient clinics in the subdivisions were excluded from the study. 

Before their appointment at a pediatric outpatient clinic, the parents or caregivers of the child in the waiting room were asked to complete the questionnaire. The parents who applied to general pediatric outpatient clinics were consecutively selected for our study. If a child was accompanied by more than one parent, only one parent was asked to participate. The questionnaires were completed without assistance, unless requested. During the study period, parents over 18 years of age were voluntarily included. The present study was approved by the Clinical Research Ethics Committee of Gulhane Training and Research Hospital (Decision Number: 2021/62). Permission was obtained from the three centers where the study was conducted.

### 2.2. Variables

To examine this, we used the Parents’ Attitudes Toward Childhood Vaccines (PACV) scale and Attitudes Toward COVID-19 Vaccine (ATV-COVID-19) scale. The questionnaire for the study consisted of a 15-item PACV, a 9-item attitude scale about the COVID-19 vaccine, demographic questions, self-report questions about COVID-19 vaccination status/intention, and vaccine-specific concerns about side effects. The questionnaire was adapted after a pilot study with 15 parents/caregivers who met the inclusion criteria. 

The questionnaire was divided into six parts. The first part consisted of seven questions on sociodemographic and economic factors (age, sex, occupation, marital status, monthly income, education level, and social security). In the second section, parents were questioned about where their children received their childhood vaccinations, whether there were any delays or vaccinations that were missed, and whether they needed any information. In the third section, the participants were asked if they had ever been vaccinated for COVID-19, had a family history of death as a result of COVID-19 infection, if they had received the COVID-19 vaccine, and their preferences for and side effects from the COVID-19 vaccine. The fourth section consisted of seven questions about social media use and the influence of social media on parents’ decisions to have their children vaccinated.

In the fifth part, the PACV scale developed by Opel et al. was used [10]. Its validity and reliability adaptation to Turkish were performed by Çevik et al. [11]. The floor and ceiling effects of the scale were within the desired limits. The Cronbach‘s alpha value of the scale was 0.676, the test–retest results were good (ICC: 0.93, p: 0.001), and the scale was found to be discriminant according to the validity of the known groups [10]. 

The PACV is a 15-item questionnaire divided into 3 domains: behavior (2 items), safety and effectiveness (4 items), and general attitude and trust (9 items). In PACV, there are three response options: binary, 5-point Likert scale, and 11-point scale (e.g., from “0, I am not quite sure” to “10, I am quite sure”). All responses were assigned a numerical value. In evaluating the scale, 2 points were awarded for hesitant responses, 1 point for “I do not know, or I am not sure,” and 0 points for non-hesitant responses; the participants were categorized as “non-hesitant” with a score of <50 and “hesitant” with a score of ≥50, which is consistent with previous research. 

The sixth and final part was the Attitudes Toward COVID-19 Vaccine Scale (ATV-COVID19), which was first developed by Geniş et al. (2020). [12]. This scale also has nine items and consists of two domains: positive (4 items) and negative (5 items). The statements in the scale were evaluated on a 5-point Likert-style scale; the statements include: “I strongly disagree (1)”, “I do not agree (2)”, “I neither agree nor disagree (3)”, “I agree (4)”, and “I strongly agree (5).” A value between 1 and 5 can be obtained by dividing the total score obtained by the sum of the item scores in the scale subdimension by the number of items in that subdimension. High scores from the positive attitude subdimension indicated that the attitude toward vaccination was positive. In the original study, two subdimensions (positive and negative attitudes) were used; however, in this study, only one subdimension is evaluated.

### 2.3. Statistical Analysis

The descriptive statistics of the data obtained from the study have been reported with the mean and standard deviation for the numerical variables and with a frequency and percentage analysis for the categorical variables. To compare the values obtained from the scales, the *z*-test was used for a quantitative analysis across the two groups, and an analysis of variance was used for categorical variables with three or more groups. The Tukey multiple comparison test was used to determine the difference between the groups as a result of the analysis of variance. Analyses were performed using the SPSS 22.0 statistical package (IBM Co., New York, NY, USA). The significance level was set at *p* < 0.05.

## 3. Results

A total of 1087 participants were included in the study (1276/1087 = 85.2%), and parents under 18 years of age (11) and participants with missing markers on the scales (178) were excluded (Table 1).

### 3.1. Sociodemographic Data

Of the participants, 779 (72.1%) were female and 308 (27.9%) were male. The mean age of the participants was 33.66 ± 9.1 years (minimum–maximum: 18–64). Of the participants, 993 (92.18%) were married and 358 (33.52%) were housewives. A total of 327 (30.63%) of the participants had a university degree, while 363 (33.9%) had a high school diploma. The income of 414 (38.14%) of the participants was TRY 4000 or less, and 28.21% ıwere between TR 4000 and 6000; there were 117 (10.97%) participants with the highest household income of TRY 10,000 and above (Table 1).

### 3.2. Hesitancy towards Childhood Vaccinations

Of the participating parents, 958 (89.78%) had their children vaccinated at the Family Health Centers. There were 108 (10.14%) participants where social media influenced their decision about childhood vaccinations, and 161 (15.10%) participants were affected by COVID-19 vaccine decisions. The participants were most likely to use Facebook (n = 380; 44.03%) for vaccination information. In addition, 38 (3.56%) of the participating parents postponed or did not have their children vaccinated and 761 (70.14%) parents indicated they needed more information about childhood immunizations (Table 1).

### 3.3. COVID-19-Related Variables

A total of 408 (37.92%) of the participants and 504 (46.93%) of their family members were infected with COVID-19. In addition, 105 (9.79%) participants lost one of their family members to COVID-19 infection, and 882 (82.12%) participants who were vaccinated against COVID-19 preferred the mRNA vaccine. Approximately half of the participants (n = 519; 51.08%) were exposed to COVID-19 vaccine side effects (Table 1).

### 3.4. PACV Scale

**Figure 1:**
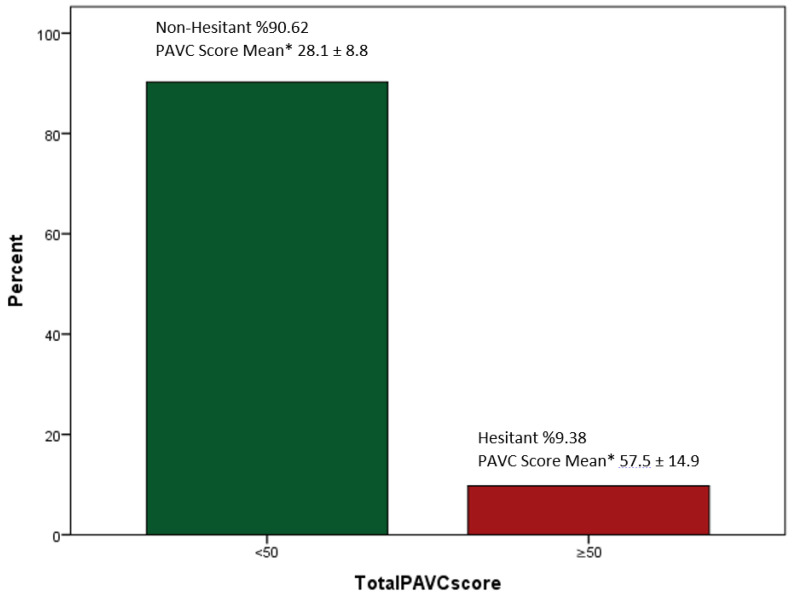
Participant Parent Attitudes about Childhood Vaccine (PACV) Scores Non-Hesitant/Hesitant. Mean ± SD: Average, standard deviation.

The average PACV score was 30.96 (SD 12.9), and the distribution of scores is shown in Figure 1. VH was noted in 102 (9.38%) parents. There was no significant difference between the age group, gender, education, or income level of the participants. Although there was no statistically significant difference, those who are not hesitant were mostly high school graduates (n = 341; 35.08%), and those who are hesitant were most often college graduates (n = 39; 38.24%). Those who were influenced by social media regarding their vaccination decisions were more hesitant (n = 27; 26.73%) than those who were not hesitant (n = 134; 13.8%) (*p* < 0.001). Those who used Facebook for vaccination information were less hesitant than those who used other social media (*p* < 0.001). On the other hand, those who used Instagram have more VH (56.16%). Those who postponed vaccinating their children were more hesitant (7.92%; <0.013) (Table 2).

### 3.5. Attitudes toward the COVID-19 Vaccine (ATV-COVID-19)

When the participants’ ratings on the Attitudes Toward the COVID-19 Vaccine scale were analyzed, the mean score for a positive attitude was 3.69 ± 0.95. Although there was no significant difference in positive attitudes toward the COVID-19 vaccine between age groups, the positive attitude scores of males were higher than those of females (*p* < 0.022,). When comparing marital status, education level, and monthly income, the mean values of the positive attitude scales did not differ. Those who had a family member with a COVID-19 diagnosis (3.77 ± 0.89) had higher positive attitude scores than those who did not (3.62 ± 0.99) (*p* < 0.007). There was no significant difference between the mean scores of positive attitudes among those who had received the COVID-19 vaccine, who had experienced COVID-19 vaccine side effects, and those who had lost a family member to COVID-19. In addition, there was no difference between the positive attitudes of parents who had or had not deferred their childhood vaccinations (Table 3).

### 3.6. PACV Scale and ATV-COVID-19

The participants who indicated that social media did not influence their decision to get their child vaccinated had higher positive scores than those who indicated that it did (*p* < 0.001). Among the social groups, the most positive attitude was found on Facebook (3.97 ± 0.46), (*p* < 0.001). A statistically significant difference was found between the positive attitudes of parents who were hesitant and not hesitant about childhood vaccines (*p* < 0.001). Parents who are hesitant to childhood vaccinations (2.84 ± 0.97) have lower positive attitudes towards the COVID-19 vaccine than those who are non-hesitant (3.77 ± 0.9).

## 4. Discussion

This is the first multicenter study with a large sample using the PACV scale to determine VH in Turkey. Our study has shown that parents who were hesitant about childhood vaccines also displayed less positive attitudes toward the COVID-19 vaccine. In addition, VH, as identified by a high PACV score, was associated with non-vaccination or vaccination delay. The present study showed that people influenced by social media had hesitant attitudes toward childhood vaccines and less positive attitudes toward COVID-19 vaccines when making decisions about childhood vaccines and COVID-19 vaccines. In addition, the presence of a COVID-19 diagnosis in the family led to a positive attitude toward the COVID-19 vaccine, whereas the absence of such a diagnosis was associated with VH.

In the current study, the hesitancy toward childhood vaccines did not change based on age, education, and income level. A study in Turkey found that women who lived in developed regions and had a higher income and higher education levels were more hesitant and more likely to refuse vaccination [13].

In our study, 9.38% of participants are VH. In the literature, the prevalence of VH in PACV varied from 5% to 34.7% in different countries (using the conventional PACV cut-off) [14,15]. Hence, there is a need to conduct studies at different time points in different regions to predict vaccination hesitancy in Turkey. 

We found that hesitant parents were more likely to delay or not vaccinate their children. The literature has shown that parents with a PACV score of ≥50 were at higher risk of postponing or not having their children immunized [16,17]. It has been noted that hesitancy around measles vaccination, as identified by PACV in Sudan, was found to have a direct impact on measles vaccine administration [18]. Screening parents who are hesitant to vaccinate can be done with the PACV and may be a useful tool that can be used to support vaccination throughout childhood.

Another finding of the present study was that women have less positive attitudes toward the COVID-19 vaccine than men. In studies of COVID-19 vaccines, it has been observed that men were more willing to be vaccinated [19,20,21]. Research has shown that women in Turkey were hesitant toward the COVID-19 vaccine [13,19,20]. 

Parents whose families had not been diagnosed with SARS-CoV-2 infection were more reluctant to have their children vaccinated. In their study, Goldman et al. showed that parents’ intention to vaccinate their children against influenza increased after the COVID-19 pandemic [21]. Another study from Indonesia found that knowledge of Zika was significantly associated with lower vaccination compliance among children [22]. Similarly, Opel et al.’s study supported the hypothesis that the pandemic may have positively influenced parents’ general attitudes toward childhood vaccination [23]. In our study, when a family member was diagnosed with SARS-CoV-2, positive attitudes toward COVID-19 vaccines were higher than among those who did not have the disease.

In India, Mohan et al. showed that parents with SARS-CoV-2 had more positive attitudes toward COVID-19 vaccines [24]. One of the many international studies covering all 27 EU member states showed that vaccination readiness was lower among people who tested positive for SARS-CoV-2 than among those who had not [25]. Our study is in line with the findings of Mertens et al., who found that increased anxiety during the COVID-19 pandemic was associated with perceived risks to family members and health anxiety [26]. Infection of relatives of parents may have positively affected parents’ attitudes toward vaccines by increasing their perception of the value of vaccines in preventing communicable diseases.

Our study found that those who reported being influenced by social media regarding their children and their vaccination decisions were more hesitant about routine childhood vaccinations than those who were not. Accessing vaccine-critical websites within 5–10 min has been shown to increase perceptions of vaccination risk, decrease perceptions of risk of not being vaccinated, and decrease the intentions to be vaccinated [3]. 

Parents not influenced by social media for the COVID-19 vaccine have been found to have more positive attitudes toward vaccination. Along with the pandemic, many studies on this topic have supported our work [25,26,27,28]. 

Those participants who used social media for vaccine information mainly used Facebook and Instagram. Facebook was the most used social media platform by parents who were not hesitant about childhood vaccines and who had a positive attitude toward the COVID-19 vaccine in this study. In their study of social media use and influenza vaccination among adults, Ahmet et al. found that those who used Facebook as a source of health information were more likely to be vaccinated [29]. Facebook is a powerful platform for finding and sharing health-related content and providing social support [30]. This may be because of the ban on anti-vaxx ads on Facebook, which have prevented the spread of vaccines and resulted in vaccine misinformation [31]. This question can be answered by a new study that examines the factors that help explain the content of the vaccination discourse on Facebook pages, along with what drives and generates that discourse.

Instagram is the most widely used social media for those hesitant about childhood vaccinations. Some posts have described some vaccine-related side effects and the harms of the vaccine, but these were usually presented with a narrative and visuals, thereby triggering anxiety in parents [32]. They continued to spread antivaccine sentiments by creating new antivaccine tags to replace closed accounts or by using pro-vaccine tags. With recent changes, posts on Instagram can now be reported for “false information” [33].

Another important finding of our study was that 70% of the parents reported that they needed information about vaccines. In various studies conducted with parents in Turkey, a lack of knowledge was cited as a cause of incomplete vaccinations and postponement of vaccinations [34,35]. Similar to our study, in the Canadian Immunization Research Network survey, 68% of parents with 24–59-month-old children across Canada reported that they frequently needed to research or obtain information about vaccines [36]. In the future, it is important to meet the need for information to prevent the escalation of vaccination refusal and denial [3].

Parents who are VH, as determined by the PACV scale, have a less positive attitude toward the COVID-19 vaccine. In their study about the acceptability of COVID-19 vaccination among children in India, Mohan et al. found that parents whose children were routinely vaccinated were willing to have their children vaccinated against COVID-19 [24]. This was also noted by Altulaihi et al., who reported that parents who were very receptive to seasonal influenza vaccination were also willing to accept the COVID-19 vaccine for their children [37]. El-Elimat et al. found that participants who had received an influenza vaccination were more likely to accept the COVID-19 vaccine compared to those who had not received an influenza vaccination [38].

The present study has shown that, in Turkey, the parental attitude toward childhood vaccination and use of social media leads to hesitant and uncertain vaccination decisions and results in parents needing information about vaccinations. Developing continuing education programs that provide information for health professionals to appropriately address concerns about vaccination, as well as communication strategies to help them confidently recommend vaccinations, will support the efforts in this area.

One of the strengths of the present study was the use of previously approved survey instruments (PACV and ATV-COVID-19) to determine parental hesitancy toward vaccination. In addition, the large sample size was one of the strengths of our study.

A possible limitation was the current study’s nature: the participants were in hospitals, which may have increased the potential for higher vaccine acceptance. In addition, it is possible that those individuals who were hesitant at the time of participant selection may have been less likely to participate in the survey. This was a cross-sectional survey that provided information or shed light on the situation in a snapshot, and the timing of the cross-sectional snapshot may be unrepresentative of the behavior of the group as a whole. This means that parents who do not regularly see providers or have access to healthcare may not be included, thereby creating selection bias; hence, the results may not be representative of Turkey as a whole.

However, the sociodemographic data of the participants in this study show similarities with the Turkish population. According to 2018 data from the Institute for Population Studies at Hacettepe University, one-third of women and men in the Turkish population have a primary school degree, and 26% of women and 33% of men have a high school degree, which is similar to the participants in our study [39]. The average age of the Turkish population is 33.1 years, which is similar to that of our participants [40]. Furthermore, since there is no referral chain in Turkey, patients can contact hospitals directly. In addition, 76.7% of the population prefers state hospitals for medical care [41]. For these reasons, our study can provide helpful information about VH in Turkey.

## 5. Conclusions

Vaccine hesitancy is a serious threat to effective vaccination programs against COVID-19 and childhood vaccination. In this study, parents who were hesitant about childhood vaccines also showed a less positive attitude toward COVID-19 vaccines. Our analysis shows that VH and attitudes toward COVID-19 vaccines were influenced by social media use. These findings suggest that public health interventions that use social media may be effective in promoting vaccine uptake and preventing hesitant attitudes toward vaccination in Turkey. Furthermore, parents need information about vaccinations. A total of 89.78% of participating parents had their children vaccinated at family health centers, and we recommend the development and use of health education about vaccinations and vaccine-preventable diseases here. Considering these findings, the national government and professionals, pediatricians, and public health experts must implement reliable strategies to address the detrimental effects of misinformation, which can escalate vaccine hesitancy. In addition, it is important to study the factors that contribute to vaccine hesitancy in different Turkish regions. The controversy surrounding the COVID-19 vaccines may diminish parents’ confidence in routine childhood immunizations. Understanding the complex causes of vaccine hesitancy can help public health policy overcome barriers and increase vaccination rates.

## Figures and Tables

**Table 1:** Descriptive Characteristics of the Participants.

		N (1087)	%
Average age 33.21 ± 8.9			
Age group	<35	587	54.97
	≥35	481	45.03
Gender	Male	298	27.9
	Woman	770	72.1
Marital status	single/separated/divorced	84	7.82
	Married	984	92.18
Level of education	Literate/primary/second	300	28.12
	High school	363	33.99
	University	327	30.63
	MSc/PhD	78	7.26
Job	Housewife	358	33.52
	Employee	113	10.61
	Officer	167	15.59
	Farmer	269	25.16
	Other	161	14.66
Social Security	Pension fund	150	14.03
	SSK	480	44.9
	Bagkur	320	29.93
	Private insurance	28	2.62
	Non	80	7.48
	Other	11	1.03
Family’s monthly income	TRY 4000 or less *	407	38.14
	TRY 4000–6000	301	28.21
	TRY 6000–8000	150	14.06
	TRY 8000–10,000	92	8.62
	TRY 10,000 and above	117	10.97
**Assessment of Childhood Vaccine Hesitancy**			
Place application of childhood vaccination	Family health center	958	89.78
	State Hospital	35	3.28
	University Hospital	7	0.66
	Private hospital	21	1.97
	Private practice	1	0.09
	multicenter	45	4.22
Need more information about childhood vaccines		761	70.14
Delayed or missed to child’s vaccinations		38	3.56
**Social Media Related Variables**			
Social media for vaccine information	İnstagram	266	30.82
	Twitter	74	8.57
	Facebook	380	44.03
	WhatsApp	40	4.63
	Other	103	11.94
Social media influenced childhood vaccines		108	10.14
**COVID-19-Related Variables**			
Infected with COVID-19		408	37.92
Family member/s being infected COVID-19		504	46.93
Dead family member/s because of COVID-19		105	9.79
Vaccinated with COVID-19 vac.		882	82.12
Side effects related to the COVID-19 vaccine		519	51.08
Social media influenced decisions about the COVID-19 vaccine		161	15.10

* 2021 minimum wage was 4250 TL. SSK. Social Insurance Institution Bağkur. Social Security Organization for Artisans and the Self-Employed MSc Master of Science, PhD Doctor of Philosophy.

**Table 2:** The Relationship between Parental Attitudes about Childhood Vaccines and Characteristics of Participating Parents.

Average Score Mean ± SD (Min–Max)		30.96 ± 12.93 (0–87)				
		PACV Group				*p*
		Non-Hesitant		Hesitant		
		N	%	n	%	
Total		985	90.62	102	9.38	
Age group	<35	517	55.65	47	48.45	0.299
	≥35	412	44.35	50	51.55	
Gender	Male	277	28.67	21	20.59	0.083
	Woman	689	71.33	81	79.41	
Marital status	Single/separated/divorced	76	7.82	8	7.84	0.993
	Married	896	92.18	94	92.16	
Level of education	Literate/primary/secondary	272	27.98	30	29.41	0.102
	High school	341	35.08	24	23.53	
	University	290	29.84	39	38.24	
	Master’s/doctorate	69	7.10	9	8.82	
Family’s monthly income	TRY 4000 or less	378	39.13	29	28.71	0.204
	TRY 4000–6000	273	28.26	28	27.72	
	TRY 6000–8000	132	13.66	18	17.82	
	TRY 8000–10,000	81	8.39	11	10.89	
	TRY 10,000 and above	102	10.56	15	14.85	
Delayed/missed child’s vaccinations	Yes	30	3.10	8	7,92	0.013
	No	937	96.90	93	92.08	
Infected with COVID-19	Yes	597	61.29	71	69.61	0.100
	No	377	38.71	31	30.39	
Family member/s infected with COVID-19	Yes	504	51.80	66	65.35	**0.009**
	No	469	48.20	35	34.65	
Dead family member Due to COVID-19	Yes	877	90.32	91	89.22	0.721
	No	94	9.68	11	10.78	
Vaccinated with COVID-19 vaccine	Yes	181	18.60	11	10.89	0.054
	No	792	81.40	90	89.11	
COVID-19 vaccine side effect	Yes	470	51.14	49	50.52	0.906
	No	449	48.86	48	49.48	
Impact of social media COVID-19 vaccine	Yes	134	13.89	27	26.73	**0.001**
	No	831	86.11	74	73.27	
Social media for vaccine information	İnstagram	225	28.48	41	56.16	0.001
	Twitter	66	8.35	8	10.96	
	Facebook	376	47.59	4	5.48	
	WhatsApp	38	4.81	2	2.74	
	Other *	85	10.76	18	24.66	
Impact of social media child’s vaccination	Yes	88	9.13	20	19.80	**0.001**
	No	876	90.87	81	80.20	

**Table 3:** Descriptive Statistics and Determination of the Factors that Affect Positive Attitudes Based on Demographic Characteristics.

		COV_19_ Positive Attitude	
		Mean * ± SD	*p*
TOTAL		3.69 ± 0.95	
Age group	<35	3.69 ± 0.93	0.615
	≥35	3.72 ± 0.93	
Gender	Male	3.79 ± 0.83	0.022
	Woman	3.65 ± 0.98	
Marital status	Single/separate/divorced	3.65 ± 0.98	0.691
	Married	3.69 ± 0.94	
Level of education	Literate/primary/secondary School	3.69 ± 0.9	0.491
	High school	3.69 ± 0.9	
	University	3.64 ± 1	
	Master’s/PhD	3.82 ± 1.14	
Family’s monthly income	TRY 4000	3.73 ± 0.9	0.644
	TRY 4000–6000	3.67 ± 0.96	
	TRY 6000–8000	3.6 ± 0.94	
	TRY 8000–10,000	3.69 ± 0.97	
	TRY 10,000	3.67 ± 1.09	
Delayed/missed child’s vaccinations	Yes	3.53 ± 0.95	0.302
	No	3.69 ± 0.95	
Infected with COVID-19	Yes	3.75 ± 0.91	0.081
	No	3.65 ± 0.96	
Family member being infected with COVID-19	Yes	3.77 ± 0.89	0.007
	No	3.62 ± 0.99	
Dead family member because of COVID-19	Yes	3.6 ± 1.07	0.286
	No	3.7 ± 0.93	
Vaccinated with COVID-19 vaccine	Yes	3.69 ± 0.97	0.866
	No	3.68 ± 0.83	
COVID-19 vaccine side effect	Yes	3.67 ± 0.99	0.849
	No	3.71 ± 0.87	
Impact of social media COVID-19 vaccine	Yes	3.48 ± 1.12	0.002
	No	3.73 ± 0.91	
Social media platform for vaccine information	İnstagram	3.51 ± 1.09 ^b^*	0.001
	Twitter	3.46 ± 1.13 ^b^	
	Facebook	3.97 ± 0.46 ^a^	
	WhatsApp	3.23 ± 1.15 ^b^	
	Other	3.57 ± 1.04 ^b^	
Impact of social media child’s vaccination	Yes	3.45±1.07	0.007
	No	3.71 ±0.92	
PACV group	Non-Hesitant	3.77 ± 0.9	0.001
	Hesitant	2.84 ± 0.97	

Mean ± SD: Average, standard deviation. * *p* < 0.05; Analysis of variance; ^a,b^ Different letters represent the difference between groups (Tukey test). * YouTube, Google, Web, etc.

## Data Availability

Not applicable.
